# A Comprehensive Revision of Radiation Immunotherapy and the Abscopal Effect in Central Nervous System Metastases: Reassessing the Frontier

**DOI:** 10.3390/cimb46100658

**Published:** 2024-10-02

**Authors:** Júlia Moscardini-Martelli, Alejandro Rodríguez-Camacho, Jorge Alejandro Torres-Ríos, Juan Marcos Meraz-Soto, José Guillermo Flores-Vázquez, Laura Crystell Hernández-Sánchez, Francisco Javier Lozano-Ruiz, Federico Maldonado-Magos, Dharely Cid-Sánchez, Christian Haydeé Flores-Balcázar, Miguel Ángel Celis-López, Guillermo Axayacatl Gutiérrez-Aceves, Fabiola Flores-Vázquez, Sergio Moreno-Jiménez

**Affiliations:** 1 Radioneurosurgery Unit, National Institute of Neurology and Neurosurgery Manuel Velasco Suárez, Mexico City 14269, Mexico; juliamoscardini@outlook.com (J.M.-M.); j.alejandro.torres.rios@gmail.com (J.A.T.-R.); marcosmerazcx@gmail.com (J.M.M.-S.); jose.fv@outlook.es (J.G.F.-V.); dralaurahernandez33@gmail.com (L.C.H.-S.); ma.celis@innn.edu.mx (M.Á.C.-L.); neuroaxa@gmail.com (G.A.G.-A.); smoreno@innn.edu.mx (S.M.-J.); 2 Radiotherapy Service, National Cancer Institute, Mexico City 14080, Mexico; dr.lozanoruiz@icloud.com (F.J.L.-R.); maldonadofederico_1108@yahoo.com.mx (F.M.-M.); dhare_227@hotmail.com (D.C.-S.); christian.floresb@incmnsz.mx (C.H.F.-B.); 3 American British Cowdray Medical Center, Cancer Center, Mexico City 01120, Mexico; fabfloresvazquez@yahoo.com.mx; 4 American British Cowdray Medical Center, Neurological Center, Mexico City 01120, Mexico

**Keywords:** abscopal effect, immunotherapy, radiotherapy, radiosurgery, oncology, metastasis

## Abstract

Seventy years ago, Robin Mole introduced the concept of the abscopal effect to describe a rare phenomenon. This occurs when local radiation triggers an immune-mediated reduction in tumors outside the treated area but within the same organism. Observing this effect has been linked to improved overall and progression-free survival in patients who experience it. While the abscopal effect was once considered rare, it is now being observed more frequently due to the combination of radiation with immunotherapy. As a result, more researchers are exploring this study area, which shows promise for excellent results. This review focuses explicitly on the immunological implications of activating the abscopal effect through ionizing radiation in the central nervous system and explores the potentially involved immunological pathways.

## 1. Introduction

Brain metastases, the most common intracranial tumors in adults, arise primarily from lung, breast, renal, and colorectal cancers and melanomas, with approximately 200,000 new cases diagnosed annually [1,2,3]. The treatment of these metastatic tumors is challenging, where radiotherapy (RT) has been established as effective but is often limited by toxicity and efficacy concerns. The discovery of the abscopal effect—where localized RT induces immune-mediated antitumor responses beyond the irradiated area—presents a significant opportunity to enhance treatment outcomes. Understanding the abscopal effect and its relationship with immunotherapy opens a new possibility for the way of conceiving metastasis treatment.

RT utilizes advanced 3D imaging to deliver precise radiation doses to tumors while minimizing damage to surrounding healthy tissue, leading to tumor cell DNA damage and subsequent cell death. The concept of the abscopal effect was first introduced in 1953 by Mole, indicating that local radiation can provoke systemic immune responses, either partial or complete, based on antitumoral activity, increased activation of dendritic cells, and stimulation of CD8 T lymphocytes and NK cells. The term “abscopal” derives from the Latin ab, meaning “position away from” and Scopus, meaning “mark” or “target” [4,5,6,7,8]. Other proposed theories about the abscopal effect include increased tumor receptors, changes in cytokine patterns, and altered functioning of effector T cells [7,8,9,10,11,12]. Related concepts include the “bystander effect”, where non-irradiated cells in proximity to irradiated cells respond to signals sent from those cells and the “cohort effect” at heterogeneous radiation fields in which high-dose irradiated cells may send signals that affect low-dose irradiated cells and vice versa [13].

Factors influencing the likelihood of an abscopal response include myelosuppression and previous radiation or chemotherapy, which can diminish lymphocyte availability. The high incidence of central nervous system (CNS) metastases in cancer patients presents a significant clinical challenge, despite advancements in systemic therapies and RT, the management of CNS metastases remains complex, necessitating a thorough exploration of innovative treatment strategies. This review aims to provide a comprehensive synthesis of the existing knowledge regarding radiation immunotherapy and the abscopal effect, particularly in the context of CNS metastases. By identifying key insights, current limitations, and future directions in research, we hope to lay a robust foundation for the development of innovative therapeutic strategies that could ultimately improve the prognosis for patients with CNS metastases [14,15].

## 2. Methods

We reviewed the published data related to the abscopal effect, its immunological basis, and its relationship with immunotherapy, emphasizing brain metastasis. Articles were researched on PubMed, Cochrane Library, and Clinical Trials using the following keywords: abscopal effect, radiation therapy, radiotherapy, radiosurgery, stereotactic body radiotherapy, stereotactic ablative radiotherapy, brain metastasis, and checkpoint inhibitors. We limited our search to English literature and included studies that met the following criteria: the study was a clinical trial, cohort study, case series, or review article; the study investigated the abscopal effect in patients with brain metastases; and was published in a peer-reviewed journal.

### 2.1. Immunological Basis behind the Abscopal Effect

**Figure 1:**
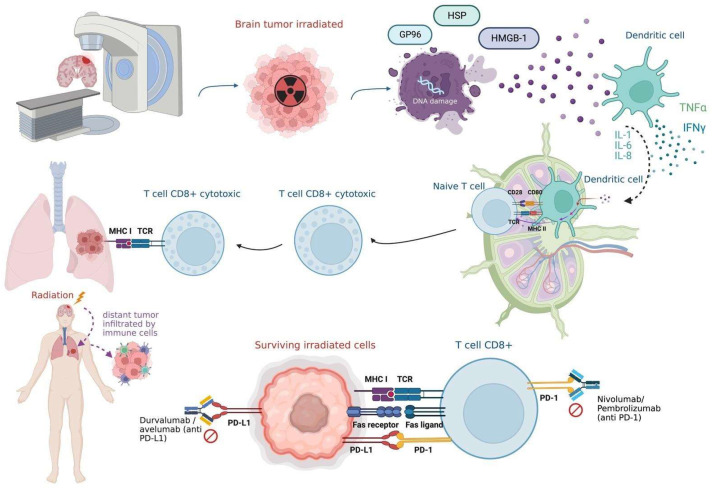
Immunologic basis of abscopal mechanisms. Following irradiation of the brain tumor, the DNA is broken into double-stranded structures, leading to IFN production. IFN triggers the sting pathway in dendritic cells and promotes anti-tumor CD8+ T cells in the lymph nodes. Furthermore, once exposed to radiation, tumor cells release antigens that are taken up by dendritic cells, which migrate to the lymph nodes and trigger naive T cells, which convert into cytotoxic T cells with the ability to migrate from the lymph nodes to distant tumor sites. Radiation-induced immunogenic cell death leads to the production of heat shock proteins (HSP), high-motility group box 1 (HMGB1), glucose-regulated protein 96 (GP96), and damage-associated molecular patterns (DAMPS), which increase dendritic cell activation through the toll-like receptor 4 (TLR4) pathway. Surviving irradiated cells increase the expression of death receptors, such as Fas, allowing an increased recognition by antitumor CD8+ T cells. Cancer cells express PD-L1 ligands, activated T cells contain programmed death receptor 1 (PD-1), and the PD-1 receptor interacts with PD-L1, enabling tumor cells to avoid recognition by the immune system. It is possible to enhance the reaction of the abscopal response by combining radiation and monoclonal antibodies of checkpoint inhibitor therapy. Created with BioRender.com.

Radiation has important indirect and direct effects according to the kind of therapy utilized, with DNA damage being the main mechanism implicated in radiation therapy and hydrolysis-induced free radicals and endothelial damage being the main effects related to radiotherapy [16]. In addition to causing cytotoxic effects in tumor cells, radiation induces effects in the immune system, resulting in an immune-mediated antitumor response [16]. The release of damage-associated molecular patterns, brought on by radiation damage in the tumor cell, immunizes the host. As a result, immune effector cells may become widely activated, and they may then attack tumor cells located miles away from the irradiation site [17]. It has been suggested that the abscopal phenomenon is due to a systemic immune response in which the tumor microenvironment is reprogrammed by local radiation, thereby generating a re-oxygenation in hypoxic tumors, altering the expression of hypoxia-inducible factor 1a (HIF-1a) levels, affecting the mechanism of angiogenesis, and leading to a critical antitumor response [18]. A high radiation dose has been shown to induce enhanced antitumor activity with increased antigen presentation by dendritic cells. Radiation induces the rupture of DNA from tumor cells, and the broken strands that remain in the cytosol activate the sting pathway, triggering the production of IFN and cytokines that promote the activation of dendritic cells and natural killer cells. The dendritic cells then migrate to the lymph nodes and stimulate CD8+ T lymphocytes, acquiring the ability to differentiate into cytotoxic T lymphocytes that migrate to the tumor sites and attack the tumor [7,9,10]. In addition, the surviving irradiated cells become more susceptible since they increase the expression of death receptors, which allows specific CD8+ T cells to recognize and eliminate these tumor cells more effectively [18] (Figure 1).

Abscopal is considered a rare occurrence. An increasing number of reports of the abscopal effect are seen where radiation therapy is combined with various immunotherapeutic agents that enhance the immune response or remove the inhibitory effect of existing immune checkpoints [16].

However, a few cases in which RT was used alone have been documented to produce an abscopal effect. When observed in cases treated with radiotherapy alone, the abscopal effect is seen independently from treatment dose, fractionation, modality, and the characteristics of the target lesion. Most of the instances that were documented were lymphoma, melanoma, and renal cell carcinoma [16].

Moreover, patients with a heavy tumor burden have a lower chance of experiencing an abscopal response than those with a light disease burden. Hence, it is more likely to achieve an abscopal response at slight baseline disease volumes [19,20]. Thus, an abscopal reaction is also thought to be related to a smaller treatment field. Wider treatment fields expose more T cells to radiation, exhausting them and preventing them from launching an immunological response [21].

### 2.2. Radiation Therapy Parameters

A universal consensus on the best RT settings to trigger an abscopal response has yet to be reached. According to various published information, the abscopal effect can be observed as early as the second week, with doses ranging from 0.45 Gy to 74.8 Gy [16]. According to certain statistics, single-fraction radiation is preferable to multiple-fraction radiation [22]. Single-fraction radiation and radiation with numerous fractions have similar results in several studies [23]. Other variables, such as the tumor type and radiation methods, may be to blame for the unpredictability of these results. Mackley et al. discovered that in individuals with metastatic melanoma treated with radiation treatment and anti-cytotoxic T lymphocyte-associated antigen 4 (CTLA-4), a greater biological equivalent dose was substantially related to an abscopal response [24].

### 2.3. Combining Immunotherapy with Radiotherapy to Induce an Abscopal Response

As we previously explained, when a tumor is exposed to radiation, the cellular stress or injury in the tumor may result in the liberation of neoantigens, or tumor-specific antigens, in the context of necrotic and apoptotic tumor cells. In this vein, adding immunotherapy to RT would increase this impact. Also, several immunotherapeutic drugs can enhance abscopal responses by focusing on various facets of the immune-mediated response [25]. For example, anti-CD40 can be used to increase antigen-presenting cell activation; FMS-like tyrosine kinase receptor three ligands (FLT3L) can be used to attract and stimulate antigen-presenting cells, anti-CTLA4 or antibodies against programmed cell death protein 1 (PD1) can act as immune checkpoint inhibitors, increasing the T cell activity directed against tumor cells [21,24,25,26,27].

Inducing an abscopal response with RT alone remains challenging. Combining immunotherapy with RT appears to stimulate this response, and an increasing number of investigators are developing clinical trials combining radiotherapy with multiple immunomodulators to induce an abscopal effect in the setting of brain metastases [24,28,29,30]. (Table 1)

A clinical trial by Formenti, M.D. (NCT03449238), is assessing the abscopal response in breast cancer patients with at least two brain metastases within eight weeks after using combination therapy with stereotactic radiosurgery (SRS) and pembrolizumab. Other parameters analyzed are the possible correlation between the abscopal response, radiation dose, and overall survival [28].

Khan, M.D. (NCT02858869) is leading a pilot trial studying the side effects of administering pembrolizumab in combination with SRS to treat patients with melanoma or non-small cell lung cancer with brain metastases. It is proposed that pembrolizumab may inhibit tumor cell growth and spread. The abscopal response, overall survival, local recurrence rate, and rate of symptomatic radiation necrosis are among the side effects investigated in this study [29].

Other ongoing research by Lin, M.D. (NCT03483012) at the Dana-Farber Cancer Institute is testing whether giving atezolizumab in combination with SRS can boost the abscopal response and thus enhance the effects of SRS or atezolizumab administered alone. The safety of this combination, progression-free survival (PFS), overall survival (OS), and the onset of radiation necrosis are all being assessed in this research [30].

Several studies unrelated to brain tumors have shown that RT plus immunotherapy induces the abscopal effect [20,26,31].

### 2.4. Checkpoint Inhibitors

While radiation is a cornerstone in the management of brain metastases, systemic medicines have shown poor blood–brain barrier penetration. Immune checkpoint inhibitors have recently come to light as a possible therapeutic option for people with brain metastases. Thought to be an immune-privileged area, the central nervous system was proven to be false when brain metastases were discovered to be surrounded by an inflammatory milieu. Checkpoint inhibitors have been shown to be effective in treating brain metastases, contrary to earlier theories that monoclonal antibodies could not penetrate the blood–brain barrier due to their size. Anti-tumor T lymphocytes that may be primed and activated at extracerebral locations and leaky tumor neo-vessels may be responsible for this reported activity [32]. Thus, immune checkpoint inhibitor therapy has recently increased awareness of the abscopal effect among radiation oncologists.

Lung cancer is the most common cause of brain metastases. Non-small cell lung cancer cells express the ligands PD-L1 and PD-L2, which the PD-1 receptor interacts with. Activated T cells carry the programmed Death-1 (PD-1) receptor. Because of this relationship, tumor cells are able to avoid immune system identification [33]. The anti-PD-1 ICI antibodies nivolumab and pembrolizumab restore anti-tumor immunity by inhibiting PD-1 signaling, which has been found to enhance overall survival [27,33,34,35,36,37].

The second most common antecedent of brain metastases is breast cancer. Many chemotherapy treatments have thus far been ineffective in treating metastases to the central nervous system. The monoclonal antibody trastuzumab was created in response to the identification of HER-2/neu, a receptor tyrosine-protein kinase that has proved crucial in the treatment of breast cancer [38]. Trastuzumab was ineffective against brain metastases, but the antibody–drug combination trastuzumab–emtansine showed encouraging central nervous system penetration [39]. According to the results of the HER2CLIMB trial, tucatinib, a selective HER2 tyrosine kinase inhibitor, along with trastuzumab and capecitabine, may be advantageous for patients with metastatic breast cancer who have received a lot of prior treatment [40]. In patients with metastatic triple-negative breast cancer, Sacituzumab Govitecan, an antibody that targets Trop-2 and is conjugated to SN-38, a topoisomerase I inhibitor, has been demonstrated to improve median progression-free survival and overall survival compared to chemotherapy [41].

Melanoma is the third most frequent reason for brain metastases and has a high level of radiation and treatment resistance. The efficacy of BRAF and MEK inhibitors has been shown, albeit mostly in BRAF mutation-positive patients [42]. Patients with BRAF600E mutations and brain metastases from melanoma have shown that dabrafenib lessens their symptoms [43]. Patients with advanced melanoma have demonstrated dose-dependent effectiveness with the CTLA-4 antibody Ipilimumab [44].

Dermatologic toxicity is one of the most frequent adverse effects associated with checkpoint inhibitors that patients receiving CTLA-4 or PD-1/PD-L1 inhibition have experienced. Also, frequent and more frequent in patients receiving CTLA-4 antibodies is diarrhea. Anti-CTLA-4 antibody therapy has been associated with the development of hypophysitis, which may result from the antibodies’ binding to CTLA-4 in the pituitary. Although it occurs more frequently with PD-1 monotherapy than CTLA-4 monotherapy, pneumonitis is a potentially fatal complication that can arise after ICI therapy [27,32]. It has been shown that CTLA-4 combined with fractionated radiotherapy is more likely to produce an immune-induced abscopal effect than single-dose RT [45].

Lastly, it is challenging to generalize the ideal ordering of immunotherapy and RT. The optimal method for administering ipilimumab is to combine RT with immunotherapy. Results are worse when RT is administered before immunotherapy, encouraging the use of concurrent [46]. Durvalumab has shown promise when used in conjunction with chemoradiation [45]. Further research is needed to determine when RT plus immunotherapy should be administered for a given cancer type and immunotherapy drug.

### 2.5. Radio-Immunotherapy-Related Toxicity

A significant barrier could be the systemic and overlapping toxicities from such a combination of RT and immunotherapy. Ipilimumab and anti-PD1 antibodies have been reported to develop immune-related side effects that can be severe or life-threatening. Combining radiation and immunotherapy, which affect several phases of the immune response, may make these negative outcomes more likely. Dermatological, gastrointestinal, hepatic, endocrine, and other less frequent inflammatory disorders are immune-related adverse events of concern [25,47,48].

Multiple retrospective studies have assessed the toxic effects related to radio-immunotherapy. Overall, there is no evidence of a significant increase in toxicity for patients receiving combined immunotherapy and RT compared to patients receiving either immunotherapy or RT alone [49,50,51].

## 3. Discussion

Cancer cells, stromal cells, and immunological infiltrates are all present in the tumor environment. Depending on the cell type, tumor-infiltrating cells can exhibit tumor-suppressive or tumor-promoting activities. CD8+ T cells have been linked to anti-cancer capabilities, whereas regulatory T cells and tumor-associated macrophages have been linked to pro-tumor functions. This means that radio-immunotherapy-treated cancer types with the most extensive T-cell infiltration would be more likely to have an abscopal response. Renal cell carcinoma, lung adenocarcinoma, and melanoma had the highest aggregate T-cell infiltration ratings, according to a pan-cancer examination of tumors. Lung squamous cell carcinoma, cervical and endocervical cancer, colon and rectum adenocarcinomas, and head and neck squamous cell carcinoma are other cancer forms with high aggregate T-cell infiltration scores [52,53].

Hatten et al. (2022) conducted the first meta-analysis of the abscopal effect, which included fifty studies with a total of fifty-five patients, in which patients showed tumor regression of both the target lesion and any other distant tumoral lesion after receiving treatment with RT alone or in combination with surgery, immunotherapy, or chemotherapy. Only one patient (1.8%) did not receive RT during the treatment period, resulting in an abscopal response, although he had a history of previous RT. Thirty-four (62%) of the patients received radiation therapy alone, five (9.1%) received radiation therapy in combination with surgery, four (7.3%) received chemotherapy in combination with radiation therapy, and eleven (20%) received immunotherapy in combination with RT.

The results showed that the abscopal effect was achieved in a median of three months after RT. Post-abscopal effect, new metastases developed in 16 (29%) patients, while the remaining 39 (71%) patients had stable disease during case follow-up. Overall survival was 63% after five years, and progression-free survival was 45% [9].

Knowing whether a complete response following radiation and immunotherapy is caused by the abscopal response or by the action of immunotherapy alone is challenging. A complete response may, however, be attributed to the abscopal effect in patients who receive both radiation and immunotherapy, as there is evidence that the complete response rate is higher with radiation plus immunotherapy than immunotherapy alone [17].

Biomarkers are thought to be a helpful source of knowledge for abscopal reactions. Validated biomarkers may aid in patient selection, therapy response monitoring, or the identification of the most effective immunosuppressive treatment approaches with the fewest side effects. Biomarkers (such as assessing tumor-associated macrophages, myeloid-derived suppressor cells, and T-reg cells) could be introduced into studies studying the combination of RT and immunotherapy to help design and fine-tune strategies. Further research in this area would be worthwhile to enhance the process of enhancing abscopal effects [25,54].

When only one of several lesions is exposed to radiation, an excellent antitumor immune response is likely to be restricted to lesions that resemble the one exposed. Because each irradiated lesion offers a unique opportunity for the release of distinct tumor-associated antigens and because immune activation is influenced or constrained by each organ and the environment around the tumor, irradiating each lesion within multiple tissue beds would get around the limitations of a single tumor environment interface and increase the likelihood that antitumor immunity will be activated systemically. Clinical data supporting the use of complete irradiation administered to numerous lesions in patients receiving ICI are developing, in addition to the biological justification and theoretical benefits for doing so. In patients with oligometastatic disease, a disease with a small number of metastatic lesions, treatment of all metastatic lesions, whether with surgery, RT, or other modalities, has been proven to enhance survival outcomes and decrease the risk of disease progression [55].

The primary drawbacks of the review stem from the fact that the included articles were not subjected to a biased analysis. This absence of scrutiny undermines the strength of the conclusions and the applicability of the findings. Furthermore, there was a notable absence of critical analysis regarding the presented evidence, although it offers a broad view of the phenomenon and aims to encompass the most updated information on the abscopal effect.

Immunology, radiation oncology, and genetics are constantly advancing; however, further studies are needed to determine how RT and immunotherapy can be more tolerable by better defining the dosage, optimal sequence, and modalities. Additionally, immunomodulation and predictive biomarkers play an important role in enhancing the abscopal effect [18,19], boosting the patient’s immune system response, identifying those most likely to generate the effect, and characterizing tumor immune microenvironments to predict prognosis [33,34,53,54].

## 4. Conclusions

The abscopal effect was seen as an infrequent phenomenon; however, nowadays, we can see that it is much more frequent than was thought at that time. Furthermore, the phenomenon does not occur exclusively with RT; it has also been reported in some surgical cases, although in a smaller proportion. Having better elucidated the mechanism of action of the abscopal effect and the implication of immunology has allowed research into markers looking forward to using the abscopal effect as a potential treatment target for metastasis. Also, the combination of radiation modalities with immune checkpoint inhibitor therapy has been shown to potentiate the response of the abscopal effect, and an increasing number of researchers have joined this line of investigation that promises great results.

## Figures and Tables

**Table 1:** Current clinical studies investigating the induction of an abscopal response with SRS plus immunotherapy in brain metastases.

Clinical Trial	Objective	Description	Features	Primary Outcome and Overall Objective	Significant Result
NCT03449238 [28]	To evaluate the efficacy of pembrolizumab combined with SRS in patients with metastatic breast cancer and brain metastases.	An ongoing Phase I/II interventional trial. Patients with metastatic breast cancer having at least 2 brain metastases eligible for SRS. Pembrolizumab infusion is given 4 days after SRS and continued every 3 weeks. Patients will be followed until death.	Interventional, single-group assignment, open label, 41 patients enrolled.	Tumor response for non-irradiated brain lesions at 8 weeks (RECIST 1.1). Correlation of abscopal responses with radiation dose over 1 year. Overall survival assessed for 3 years from the start of the study drug.	The trial is ongoing, so significant results are not yet available, but expected outcomes include tumor response and overall survival data.
NCT02858869 [29]	To assess the safety and efficacy of concurrent anti-PD1 (pembrolizumab) and SRS for brain metastases in melanoma and non-small cell lung cancer (NSCLC).	Phase II trial enrolling patients with melanoma or NSCLC, with 1–10 brain metastases and at least one extra-cranial lesion. Patients received anti-PD1 every 3 weeks, with SRS administered 1–2 days post anti-PD1.	Interventional, multi-arm: - Arm A: 6 Gy in 5 fx, - Arm B: 9 Gy in 3 fx - Arm C: 18–21 Gy in single fx) 25 patients enrolled (2016–2020).	Primary endpoint: Grade 3 CNS toxicity at 3 months. Secondary endpoints: Overall survival, local control, intra-cranial progression-free survival (IC-PFS), and extra-cranial progression-free survival (EC-PFS).	No grade 3 CNS toxicity reported at 3 months. Median OS was 32.8 months. 6- and 12-month OS rates were 79.1% and 67.8%, respectively. Local control at 6 and 12 months was 95.7%. Early activation of CD8+PD1+Ki67+ T cells was associated with improved outcomes.
NCT03483012 [30]	To evaluate the safety and effectiveness of atezolizumab combined with Stereotactic Radiosurgery (SRS) in patients with triple-negative breast cancer and brain metastasis.	A Phase II clinical trial testing the combination of atezolizumab, a PD-L1 inhibitor, and SRS. Atezolizumab administered every 3 weeks, with SRS beginning within 14 days after MRI.	Interventional, experimental group for atezolizumab + SRS. 6 patients enrolled, ongoing.	Primary outcome: PFS defined from the first dose of atezolizumab to progression or death due to any cause. Secondary outcome: Include extracranial objective response rate and OS.	The trial is ongoing, with results yet to be reported. The design aims to assess both safety and efficacy of combining atezolizumab and SRS in this patient population.

## Data Availability

Not applicable.
